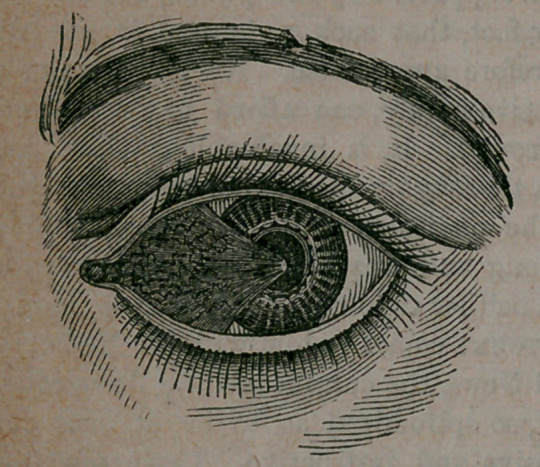# Pterygium

**Published:** 1873-04

**Authors:** 


### PTERYGIUM.

That looks like a hard word; a non-professional reader, perhaps, would be puzzled to pronounce it. But it is easy enough; call it ter-idge-ium, while we attempt an explanation of it. This is a very prevalent affection of the eye, consisting of a fleshy growth, triangular in form, starting from the inner corner of the eye, and eventually growing as far as the centre of the cornea, where it terminates in a point. It is a diseased condition of the *conjunctiva* (covering membrane), and consists of a membraneous growth, highly vascular, causing great deformity to the eye and vision, but is rarely painful.

When the eye is exposed to the wind or the irritating influences of dust or cinders, this growth becomes very livid and congested, causing a sensation as of sticks in the eye. Its appearance is well illustrated in the accompanying cut, which farmers and others, familiar with horses, will recognize as the disease common to their animals, and to which the name "hooks in the eye" is given.

In the human subject it most often occurs singly, and from the inner corner of the eye. It is frequently found double, however, growing from both corners of the eye and occasionally the upper and lower portions of the eye, as well.

These growths vary considerably in texture and color, depending somewhat upon the causes that produce them. Sometimes they are met with as a delicate white membrane,that is scarcely observed,save upon close examination. Again, they are found to be firmly attached to the eye, red and granular in appearance, and if not arrested, cover nearly the entire cornea, almost completely ruining the vision.

The causes of pterygium are somewhat obscure, and its formation very slow, years being required in which to complete their growth as far as illustrated in our cut.

It is most common among farmers, who are exposed to the heat, glare and wind of the harvest field, or to the dust of the threshing machine; these causes combining to excite congestion or inflammation in the conjunctiva, which gradually leads to thickening and enlargement of that membrane.

Miners are subjected to it from the irritation produced by particles of coal coming in contact with the eye. Furnace and rolling mill hands have the same difficulty from sparks of molten or hot iron flying into their eyes. However, whatever the cause, the effects are the same—serious impairment of the vision, if allowed to reach the cornea or clear portion of the eye.

When the pterygium is small, it is confined to the *sclerotic* or "white of the eye," and in that locality can do no harm; and, it is perhaps for this very reason that they are neglected until the cornea is reached, where, after their removal they always leave a scar, which interferes more or less with vision, depending upon the size of the attachment. When these growths are first observed, they can usually be dispersed by means of simple collyria, composed of five grains of sulphate of copper or zinc, in an ounce of rose-water, and applied to the parts twice a day with a camel's hair brush. Later, however, nothing but the most skillful surgery will reach the case.

Several means are adopted for the removal of pterygium, among the most employed of which are excision, transplantation and ligation. We do not deem it necessary to enter into an explanation of these several means of cure, as it would be impossible for the patient to avail himself of any of them, save in the hands of the ophthalmic surgeon, who is already sufficiently familiar with all the details.

Pterygium is apt to be confounded with a little yellow spot almost always present in the inner corner of the eyes of elderly people. It is a small, yellow prominence, an enlargement of the tissue under the conjunctiva, entirely harmless, and rarely increases in size, unless meddled with. Should it ever become irritable, it can be safely and easily snipped off with curved scissors.

And now, let us caution our readers against applying cauteries for the removal of the growths above described. People, especially women, are so alarmed at the prospect of being subjected to the surgeon's knife, that they are prepared to undergo almost any torture rather than to submit to a surgical operation of the simplest character.

We have actually seen persons who have applied to so-called physicians, and had nitrate of silver, in substance, applied to a pterygium, every day, with a hope of so removing it, suffering untold agony, and in some instances entirely losing their eyes, rather than to submit to an almost painless and certainly a safe surgical operation, requiring but a moment to perform, and when done, painless and effective. Any excresence or growth about the eye, no matter what its nature may be, if removed at all,should never have anything but a knife applied to it for that purpose. And this advice will hold good for operations upon all parts of the body for removal of tumors or malignant growths.

## Figures and Tables

**Figure f1:**